# Service factors causing delay in specialist assessment for TIA and minor stroke: a qualitative study of GP and patient perspectives

**DOI:** 10.1136/bmjopen-2016-011654

**Published:** 2016-05-17

**Authors:** Andrew Wilson, Dawn Coleby, Emma Regen, Kay Phelps, Kate Windridge, Janet Willars, Tom Robinson

**Affiliations:** 1 Department of Health Sciences, University of Leicester, Leicester, UK; 2 Department of Cardiovascular Sciences, University of Leicester, Leicester, UK

**Keywords:** ACCIDENT & EMERGENCY MEDICINE, PRIMARY CARE, STROKE MEDICINE

## Abstract

**Objective:**

To understand how service factors contribute to delays to specialist assessment following transient ischaemic attack (TIA) or minor stroke.

**Design:**

Qualitative study using semistructured interviews, analysis by constant comparison.

**Setting:**

Leicester, UK.

**Participants:**

Patients diagnosed with TIA or minor stroke, at hospital admission or in a rapid-access TIA clinic (n=42), general practitioners (GPs) of participating patients if they had been involved in the patients’ care (n=18).

**Data:**

Accounts from patients and GPs of factors contributing to delay following action to seek help from a healthcare professional (HCP).

**Results:**

The following categories of delay were identified. First, delay in assessment in general practice following contact with the service; this related to availability of same day appointments, and the role of the receptionist in identifying urgent cases. Second, delays in diagnosis by the HCP first consulted, including GPs, optometrists, out-of-hours services, walk-in centres and the emergency department. Third, delays in referral after a suspected diagnosis; these included variable use of the ABCD^2^ (Age, Blood pressure, Clinical features, Duration, Diabetes) risk stratification score and referral templates in general practice, and referral back to the patients’ GP in cases where he/she was not the first HCP consulted.

**Conclusions:**

Primary and emergency care providers need to review how they can best handle patients presenting with symptoms that could be due to stroke or TIA. In general practice, this may include receptionist training and/or triage by a nurse or doctor. Mechanisms need to be established to enable direct referral to the TIA clinic when patients whose symptoms have resolved present to other agencies. Further work is needed to improve diagnostic accuracy by non-specialists.

**Strengths and limitations of this study**

- Service factors causing delay between first seeking help and specialist assessment were examined through in-depth interviews with patients recently diagnosed with TIA or minor stroke.
- We also interviewed the patient's general practitioner, where relevant, enabling us to gain insights from both professional and patient perspectives.
- We did not interview other health professionals involved, such as emergency department doctors and optometrists.
- As recruitment was from secondary care, we were not able to include patients who were not referred.

## Introduction

In the UK, every year, ∼46 000 people experience a first transient ischaemic attack (TIA).^1^ Although traditionally these have been distinguished from stroke according to whether or not symptoms resolve in 24 hours, this is now recognised as arbitrary,^2^ and TIA and minor stroke are considered as a continuum of the same condition and managed in the same way.^3^ Rapid assessment and treatment of these conditions reduces the risk of early recurrent stroke. The EXPRESS study showed that a strategy of early secondary prevention initiated in an open-access specialist clinic can significantly reduce 90-day stroke recurrence following TIA.^4^ These findings were supported by SOS-TIA, where 90-day stroke rate was reduced to 1.2% from a predicted 6.0% in a hospital clinic with 24 hours access, assessing and treating patients with TIA within 24 hours of symptom onset.^5^

The National Institute for Health and Clinical Excellence recommends urgent specialist assessment for patients with TIA, using the ABCD^2^ (Age, Blood pressure, Clinical features, Duration, Diabetes) score to stratify risk.^6^ These guidelines state that patients at high-risk (score ≥4) should be assessed by a specialist within 24 hours of symptom onset, and those at lower risk within a week. Implementation of this policy requires patients to seek help urgently when they experience symptoms that may be due to stroke or TIA, and to be able to access a health professional who can apply the ABCD^2^ score and arrange timely specialist assessment.

Previous research on delay to assessment has focused on patient behaviour, particularly in initiating contact with healthcare practitioners following symptom onset. A systematic review highlighted patients’ failure to recognise the symptoms of TIA and minor stroke or to respond to them urgently by calling emergency services, as the main reasons for delays in assessment after TIA.^7^ A later review, which included patients with TIA and stroke, concluded that awareness of the significance of symptoms alone did not reduce time to presentation.^8^ Qualitative studies examining delays in seeking medical help following TIA and acute stroke have sought to understand patient decision-making. Lack of recognition or ‘denial’ of symptoms, combined with decisions to make initial contact with non-emergency services were identified as some of the overlapping factors which contribute to delays in patients accessing appropriate and timely treatment.^9–11^ The focus on patient delay factors has led to publicity campaigns designed to raise patient awareness of symptoms (eg, Facial drooping, Arm weakness, Speech difficulties and Time (FAST)),^12^ and to encourage patients to invoke emergency pathways in order to reduce time to specialist assessment.^13^

The part played by service factors in contributing to delays to specialist assessment has received relatively little attention. These represent the third (diagnostic) and fourth (pretreatment) stages of the Model of Pathway to Treatment (the earlier stages being appraisal and help-seeking).^14^ A recent qualitative study of patients with TIA found that healthcare interactions following initial contact caused additional delay. In particular, lack of available appointments and perceptions of a lack of urgency on the part of healthcare professionals (HCP) added to delay.^10^ A qualitative study of ‘stroke patients’ route to hospital also identified service factors as a reason for delay if symptoms were not initially interpreted as indicating a stroke by the health service provider (including family practitioners and emergency medical services), and could result in multiple providers being involved before the patient received the appropriate treatment.^11^ Research has also recognised the importance of an appropriate response by receptionists in general practices to recognise the symptoms of stroke and direct patients to emergency care. A study based on unannounced, simulated, patient telephone calls found that although receptionists’ knowledge of the common symptoms of stroke was generally good, they were less likely to respond appropriately to patients presenting with only one symptom or less common symptoms of stroke.^15^

In the last decades, the National Health Service (NHS) has introduced additional services to provide alternatives to traditional general practitioner (GP) and emergency department (ED) provisions. These include walk-in centres, urgent care centres and minor injuries units. These may be medical or nurse-led, and may be colocated with the ED. Additionally, telephone helplines have been established, including NHS direct (now decommissioned), and more recently, the 111 helpline. These services are targeted at people who have urgent needs that are not emergencies, and aim to increase choice, improve access and relieve pressure on EDs. However, this has created a complex system for patients to navigate, and may result in duplication of care.^16^

In this paper, we aim to understand how service factors contribute to delays to specialist assessment following action to seek help from a HCP. Interviews were undertaken as part of ‘Barriers to the Early Assessment of TIA and Stroke’ (BEATS), a mixed method study to improve the understanding of the facilitators and barriers to specialist assessment of TIA and minor stroke. Quantitative data for patients recruited from the TIA clinic, including delays occurring earlier in the pathway due to patient-related factors, have been published elsewhere.^12^

## Methods

In the quantitative part of the BEATS study, patients with a confirmed diagnosis of TIA or minor stroke (NIHSS score <8^17^), either admitted to the University Hospitals of Leicester NHS Trust stroke unit or referred to the rapid access TIA clinic were invited to participate. Eligible patients were approached by clinical or NIHR Stroke Research Network staff, and provided with a study information sheet. The sample included 132 patients recruited from the stroke unit and 294 from the TIA clinic. Participants without mental capacity to consent were excluded. Structured interviews were undertaken with these patients to quantify delays and map pathways from symptom onset to specialist assessment. During the first phase of the study, participants were asked if they would be willing to be approached again for a further, more detailed interview. Of the 342 people approached, 316 (92.4%) agreed. Recruitment took place between 1 December 2008 and 30 April 2010.

In this paper, we describe findings from a purposive subsample of patients selected from the ‘pool’ of 316 potential participants described above. The aim was to include accounts with the widest possible range of prompt/delayed presentations at the TIA clinic so as not to miss important potential contributions to delay. Team discussion, incorporating advice from clinicians, steering group members with patient/carer experience, and relevant literature, led to a list of selection criteria shown in table 1. Recruitment continued until we had included at least one person from each cell and interviews ceased to give new insights (data saturation). Those selected were invited (in writing) for a further, in-depth interview at their home to explore their experiences of care between seeking help to specialist assessment. In some cases, a relative or carer was also present at the interview. If a GP had been involved in these patients’ referral, permission was sought to interview him or her about their case; 42 patients and 18 of their GPs participated (out of 24 eligible). The sampling strategy was designed to maximise chances of uncovering issues that could contribute to delay, so diversity was sought in terms of severity of TIA/stroke, day of the week that symptoms were experienced, type of service initially consulted, age, gender, ethnicity and deprivation (Index of Multiple Deprivation^18^ derived from the postcode). Sampling responded to ongoing analysis; for instance, we actively sought participants who consulted more than one health professional before referral to an appropriate specialist when it became clear that this could cause delay. Recruitment took place between 1 December 2008 and 30 April 2010.

**Table 1:** Characteristics of 42 participants: (sampling strategy aim was to achieve at least one participant in each cell)

Characteristic	Number of participants
Time from symptoms to specialist assessment (hours)	
<3	7
3–24	6
24–48	6
>48	23
Diagnosis	
Stroke	13
TIA	29
Day of week symptoms were experienced	
Monday/Tuesday	13
Wednesday/Thursday	12
Friday	5
Saturday/Sunday	12
First healthcare professional consulted	
NHS direct	2
GP (in hours)	24
Out-of-hours GP or walk-in centre	5
A&E department	2
Paramedic (999)	3
Other (eg, optometrist)	6
Age (years)	
<60	12
60–75	21
>75	9
Gender	
Male	23
Female	19
Ethnicity	
White British, Irish or other	38
Asian, Asian British	3
Black/Black British	1
Index of multiple deprivation quintiles	
1 (most deprived)	11
2	6
3	11
4	7
5 (least deprived)	7

A&E, accident and emergency GP, general practitioner; NHS, national health service; TIA, transient ischaemic attack.

Semistructured interviews were carried out by Janet Willars PhD, an experienced qualitative interviewer with a background in psychology, using a topic guide that was drafted on the basis of existing literature and developed via discussions with the research team including members of the steering group with experience of stroke or TIA, and in response to issues emerging during qualitative and quantitative data collection. The guide (see online supplementary appendix) was used flexibly to enable in-depth exploration of unanticipated issues. Interviews were conducted in the participants’ homes and lasted about 60 min.

10.1136/bmjopen-2016-011654.supp1 Supplementary data

The interviews were audio recorded and transcribed before analysis, which started while data collection was ongoing. DC, KP, ER and KW developed provisional lists of issues that contributed to delay by intensively rereading a subsample of transcripts, summarising points made on a line-by-line basis to produce ‘open codes’, and grouping open codes into provisional categories. Disagreements were resolved through discussion. The coding frames were then applied systematically to subsequent transcripts by one of the above using QSR International's NVivo software for ease of management, and to provide an audit trail (NVivo qualitative data analysis software. QSR International Pty Ltd Version 7. 2006). Throughout this process, we searched for issues which did not fit the preliminary coding frame. This included presenting interim findings to members of a support group who had experienced stroke or TIA in a different city and asking if they could point out potential omissions. This process of constant comparison^19^ helped to ensure that the emerging understanding of issues associated with delay reflected patients’ and GPs’ perspectives rather than those of team members. Integration of qualitative and quantitative components was strengthened by ‘following threads’:^20^ important issues emerging from preliminary quantitative analysis were pursued by adjusting qualitative sampling and data collection strategies. Similarly, constant comparison enabled our interviewer to use reflections on earlier patient interviews to inform the prompts she used in the later patient and GP interviews, while maintaining mutual confidentiality (members of patient–GP dyads each knew that the other was participating, and that nothing one party said would be revealed to the other party).

The study was approved by the Leicestershire, Northamptonshire and Rutland Research Ethics Committee 2 (08/H0402/49).

## Results

Details of the sample of 42 patients are shown in table 1. Of these, 29 had a TIA and 13 had a stroke. The sample included a range of age, deprivation, delay in seeking help, and choice of which health professional was first approached. Delays due to service factors were experienced by most of the patients interviewed, and many of the 18 GPs felt that there were areas where the service factors contributed to delay. In the quotes presented, each patient was allocated a code number prefixed by ‘P’, and the corresponding GP was given the same number prefixed by ‘GP’.

Service factors that led to delays in patients receiving specialist assessment were considered in two parts of the pathway: first, delays in initial assessment following patients making contact with a service and, second, delays in specialist assessment after an initial assessment.

### Delays in initial assessment by general practice following the patient contacting the service

Several of the 24 patients who chose their GP as the first point of contact—and GPs themselves—acknowledged difficulties obtaining appointments as a delay factor. Most patients tried to see their GP ‘urgently’ but did not consider their case to be an ‘emergency’. The lack of an agreed definition of an ‘urgent’ appointment led to variable delays; some practices’ policy was to see such cases on the same day, in others the target was within 48 hours.

GP14. If they just say ‘can I have an appointment’ and they (receptionists) say ‘oh we're full, can you come down at half past four?’ and they say ‘yes’, then that's the end of it.

GP28. Only a few (patients), if we're on a particularly busy day, have to wait till the next day.

GP32. We operate an advanced type of system, usually we'll see them within forty-eight hours. So sometimes they (receptionists) might give them an appointment within twenty-four, forty-eight hours, and they just take it.

Even when a case was recognised as needing emergency assessment, as for TIA, the working pattern of general practice caused difficulties accommodating these needs, either in the surgery or at home.

GP18. In this situation I guess you're going to want to actually see the patient to assess them, and the way general practice is now, with the length of clinics we have, it's difficult if something was to come in during the middle of a clinic—because I know the latest advice is that they (TIA patients) should be assessed as an emergency, as urgently as possible, ideally within an hour. The difficulty is when you've got a clinic that's running for three hours booked in with urgent patients, it's not always possible to assess (these) patients.

GP24. I think a lot of TIAs end up being either home visits, or urgent appointments. so somebody rings up at eight o'clock and I go on as a home visit, so by the time I get there at one o'clock, it's imperfect, isn't it? Because people ring up and say ‘come and visit please’, doctors say ‘yes’; they don't question people. So often there's no history, so they can wait a long time before we get there.

GP06. Most of our home visits are at lunch time. In the morning, if something's life threatening, an emergency and we need to go out, we'll go out. I mean, somebody, if they were comfortable and fine at home, they would probably have just a routine end of surgery visit.

Patients also had varying definitions of what constituted an urgent appointment; for some this was just the next available slot.

P10. I just asked for the next available appointment with the doctor and that was Friday…

P31. And next day, Monday morning, I phoned, because that's the way we make appointments—you ring the doctor eight o'clock, between eight and half eight, you are seen on the same day. So I rang up and went to see the doctor that afternoon.

An important issue identified by patients and GPs was the contribution receptionists (and practice policies on their roles and responsibilities) could make in helping patients with TIA to be assessed urgently. There appeared to be a conflict between the principle that patient confidentiality should be maintained by receptionists not asking detailed questions, and the acknowledgement of their role in identifying urgent or emergency cases; where this balance settled varied between practices. In many cases the amount of information sought by receptionists depended on the request made by the patient and the urgency they were seeking. This was also reflected in policies described by GPs.

GP22. I'm sure receptionists do have a role. I hope they're aware, and we do have a written protocol that they would offer an urgent appointment if someone had a stroke or TIA symptom, but I guess there are going to be situations where we simply don't know that that's why they're asking for an appointment.

GP14. They'd have to say ‘I'm worried I've had a stroke’ or something like that. Or ‘I had some weakness down my arm and it’—our receptionists aren't primed to ask the patient what the problem is when they ask for an appointment, and we do that as a matter of policy really for confidentiality for the patient.

GP40. If people ask for an urgent appointment we tend to push the receptionists—we are trying to push them (so) we can fit them in with an appropriate person—but a lot of people won't say, and don't want to say. I think patient confidentiality is so precious.

One patient felt that it would have been helpful if the receptionist had asked them what their symptoms were to ensure a speedier response.

P10. When I made the initial appointment with the GP, I should have said what had happened and then perhaps the receptionist might have put me in a bit earlier so to speak. I suppose it would be useful if when you make an appointment, they ask you what it's about.

As well as allocating appointments, receptionists have a potential role in advising cases thought to be an emergency to speak to the GP or to call 999.

GP06. Often what will happen is somebody will ring up and speak to reception first and say ‘oh my wife's got, you know, funny speech, she's not quite right, she's got weakness’ and the [receptionists] will say ‘well it could be stroke, we advise you to call 999′ and often they don't take that advice over the phone from the receptionists, so then often they get put through to us and when we advise to do it, they normally do it straightaway.

GP18. She came through to reception with the symptoms of not being able to speak properly—her husband not being able to understand her sentences—so the receptionist spoke to the doctor … who suggested that they immediately phone 999 because there was a chance that it was a stroke or mini stroke.

### Delays in specialist assessment after first contact with HCP

Both patients and GPs described delays between initial HCP contact and specialist assessment following TIA and minor stroke. These were due to delay in making the diagnosis and delays in referral pathways.

#### Delays in diagnosis

Some patients experienced delay because the HCP first consulted did not make the diagnosis. One patient described how her GP had assured her that the symptoms she was experiencing were due to shingles.

P31. I just told—she knew about my shingles, and I told her—you know, I said ‘why are my arm and leg stopping? What's wrong?’ and she said ‘nothing wrong with you, because you have shingles on the left side, and that's because your nerves are weak. That would be the reason’. She said ‘just go and have some tablets and you will be fine’.

In a case presenting with visual symptoms, the GP had referred the patient to the eye clinic. It subsequently transpired that the patient had had a TIA/minor stroke, and had a history of TIA (of which the GP was unaware at the time).

P23. The fact that my symptoms could have been stroke connected, TIA connected. But didn't seem to be taken into consideration particularly, it seems to be slightly a bit worrying that they, I saw a GP he didn't particularly help, seem to latch on to that.

Reflecting on the case, the GP acknowledged that had he been aware of the patient's history of TIA he may have acted differently.

GP23. If a patient is found—maybe I was rushing—if he'd said to me ‘oh I've had this TIA in the past and I was like this, this, this, I've had this sudden visual problem’ that might have alerted me more I have to say, or perhaps I … But I'm not sure I'd do anything different, to be honest.

One GP described delay caused by failure of an optometrist to act urgently.

GP34. The optician just said ‘make a routine appointment’ or something. The optician wrote the letter. If I would have seen it I'd have got the patient in really quickly, you know, ‘attended with intermittent loss of vision of left eye, I just refer you for assessment’—yeah, that's it, they just wanted me to do a cholesterol test, that's all they wanted me to do, I think, or a cardiovascular examination.

In addition to delays occurring within general practice, GPs and patients also described delayed diagnosis in emergency departments (ED) and walk-in centres.

P24. He (ED doctor) said it's no problem, it's the medication. Go and see your GP, come off the medication, get something different. So the next morning I saw the GP.

GP24. Well, my understanding is, he actually presented to A&E, who basically sent him away. Then he came in to see my partner, who went ‘you've had a TIA’, scored him, and he was admitted. So it was a bit of a miss, somewhere along the line.

A patient who had been seen by a doctor at a walk-in centre had her symptoms misdiagnosed as carpal tunnel syndrome. The patient subsequently saw her GP who suspecting TIA or stroke, admitted her to hospital. The GP expressed concern that the patient's condition had not been diagnosed at the walk-in centre.

P06. And he got hold of my hand and he pressed here and up here and he lifted it up in the air and he said, nothing to worry about, you've got carpal tunnel syndrome.

GP06. I think she had unusual symptoms, I think it was quite a difficult diagnosis, but I think potentially, it should have been diagnosed when she presented to the walk-in centre.

One GP described the difficulties faced by GPs in deciding whether to make a putative diagnosis of TIA and refer to the TIA clinic. Mindful of the need not to ‘over-burden’ the clinic, the GP suggested that the decision to refer a patient for specialist assessment was essentially a reasoned judgement based on the balance of probabilities.

GP38. It's these vague symptoms—you know, it can often be clouded by other factors in the patient's history; they can be an anxious individual who's prone to a variety of unexplained medical symptoms, and if we react to every single one we could single-handedly squash the TIA clinic's available facilities, so we try and use some judgement in who to refer.

#### Delays/problems in referral pathways

##### Referral by GPs

Interviews with GPs revealed variation in the use of scoring systems, and the appropriate way to refer patients for specialist assessment which could potentially result in delays between first contact and specialist assessment. During interviews, some GPs referred to the ABCD^2^ score as a strategy that could potentially help decision-making for referrals.

GP18. I guess I—I know there's a scoring system and I'd try and work out, according to that scoring system, whether they were at high risk of another TIA… If I thought there was substantially high risk of another TIA, I'd consider whether or not I actually admitted them. If I didn't think they were at high risk, I'd refer them to the stroke TIA clinic.

However, reference to the use of the ABCD^2^ tool did not feature in the majority of GP accounts, and the variation in its use was illustrated by two GPs who did mention it.

GP14. I wouldn't be able to do the score off the top of my head, to be fair, of what the different criteria were. I think it's based on age, diabetes, blood pressure…I can't remember what the points are. So I would generally make a decision about referral based on clinical diagnosis rather than on a points system.

GP6. Then they get a score, you have to tick features and then they (TIA clinic) contact them and see them as necessary. … I'll be honest, I've not really used it that many times, you know, if somebody's still got symptoms and it's over 24 h then they need to go in anyway, and if the symptoms have resolved, that's normally when we'd probably use it more.

Another GP described how he would ‘over-ride’ the scoring system if the results did not indicate the need for urgent assessment but experience told him otherwise.

GP24. And if something inside me goes ‘I'm just not happy about this', then, like every other GP, I will either fix the numbers, or I will just say ‘I don't care, this one needs to be seen. There's something—I've been doing this job for a while, and I don't care what your numbers say; I want this one seen’.

Levels of knowledge about the processes for referring patients for specialist assessment also appeared to vary among GPs. Some used the TIA form which could be faxed to the TIA clinic ensuring prompt assessment while others appeared to be using other systems for referral.

GP33. Whereas the form—… you can fill it in quickly … and you can fax it over. You've got a clear referral pathway; you know what to do, you know what the response should be.

GP23. If he said ‘I've had weakness down one side last night’, I'd fill in a TIA form and fax it off and he'd be seen within forty-eight hours.

GP28. No, we don't have a form, we just sort of dictate… it goes off by choose and book.

One patient expressed dissatisfaction with delay to be seen in clinic, even when the appropriate referral pathway was used.

P23. And so I had to go to the desk on the way out, and he (GP) gave me a form to give to them and wait for an appointment. The following day … we'd had a look on the Stroke Association website … and the general consensus of opinion seemed to be that in a situation like that I should see somebody within 24 hours.

##### Referrals by ED, walk-in centres and GP out-of-hours services

Interviews with GPs and patients suggested problems and delays in referrals to specialist assessment in cases presented in settings other than general practice, including ED, walk-in centres and GP out-of-hours services. Patients initially seen and diagnosed in these settings were often referred back to their own GP to make a referral for specialist assessment, inevitably resulting in some delay. One GP and her patient described how after being seen in ED the patient had been referred back to the GP to arrange assessment, but there had been a significant delay in the paperwork coming through from ED which had delayed the assessment considerably.

P22. They (ED) kept me in overnight…they said they would make arrangements for me to have a head scan…they didn't…they said ‘Go and see your GP’. I went to my GP…she said ‘Well why didn't they do it at the hospital?’….To cut a long story short, about 3 weeks later, I got an appointment.

GP22. I think sometimes they will go to another agency who will then say ‘you need to go and see your doctor for a referral to the stroke clinic’ and actually that's a time consuming process and sometimes you don't get relevant information from the third party.. you'd have thought if someone presents to A&E with a TIA then they should be referred straight off.

Two patients described similar delays following attendances at a walk-in centre and urgent care centre, respectively. Both were advised to make follow-up appointments with their GPs, which had concerned them given their perceptions of the seriousness of their situations and need for urgent specialist assessment.

P16. So, as I say, that were on the Friday, so I went, they took me there to the walk in, I seen a doctor there and he done various things, to see about a stroke, but he wasn't sure, now that's what I say I'm not happy about because, I mean, strokes are a serious condition that needs some pretty quick attention, but he weren't sure, now as I say, this was the Friday, and they got the appointment on the Monday.

In the second case, the patient's family reported that the GP had been concerned at the delay in arranging specialist assessment as a result of the doctor at the urgent care centre referring the patient back to their GP.

P26. Well he said that we could either admit you tonight, as in at the hospital, but nothing would be done, no tests or anything would be done, so you can go home, have your dinner and then go and see your GP and get your GP to do a referral and say that your mum's had a TIA. On the Monday morning, he (GP) said ‘how could this doctor on the Sunday afternoon say that she's had a TIA, why couldn't he do the referral there and then, why leave it another day?’

Some GPs identified barriers encountered by out-of-hours doctors in referring patients for specialist assessment. It was pointed out that out-of-hours doctors did not have access to the usual referral pathways and documentation (TIA form), and so would have difficulties in referring patients.

GP40. I don't think out of hours people would find it easy to do a TIA referral either.

GP28. I don't think they have a fixed referral pathway, if it's a TIA they tend to assess how severe it is, and if it's resolving or very mild they'll probably tell them to come and see us.

One GP noted that if the patient feels better and they may not attend surgery and the GP may be unaware of the event and the need for follow-up.

GP31. I think that the problem is with the out of hours.. sometimes they (the patient) are not seen on Monday again by the GP because by then the patient is feeling completely well and they don't actually bother to go.. whether the out of hours can use separate forms, do like a red alert—because if they use the same form to the routine fax from out of hours to us every Monday morning, those are never looked at to be honest with you, they are—loads of them.

Some patients were directed to ED by their out-of-hours doctor or walk-in centre leading to further delay which could have been avoided by referral to the TIA clinic. One patient described how their pathway to the clinic included the walk-in centre and ED.

P29. We went to the reception (at walk-in centre), this was my daughter and myself at the time, and said ‘I think I might be going to have a TIA’ and they said ‘well, you know, join the queue, which is what you've got to do’, and then when the nurse came and I told her, she said ‘you've made the wrong decision, you should have rung 999 and got an ambulance to take you directly to the hospital, we don't have a doctor in the walk in centre, it's staffed by nurses only’. The accident and emergency people looked at me, heard what I had to say and said ‘you're in the wrong place, the stroke clinic, the specialist place is at (a different hospital), we'll arrange for you to go as soon as there's an available ambulance’ and that morning, they found one and they took me straight there.

## Discussion

### Main findings

Despite the ongoing FAST campaign, many patients with symptoms due to TIA and minor stroke, whether or not they have identified the cause of their symptoms, will first seek help from their own GP. Interviews with both patients and GPs illustrated the difficulties in making sure these patients are either assessed in time for referral to a specialist clinic within the recommended timeframe (which effectively means they need to be seen the same day) or advised to contact emergency services.

While most patients in our study recognised the need for ‘urgency’, this was not always interpreted as ‘same day’, and there was variation in whether practices offered same day appointments to all patients requesting an urgent consultation. GPs and patients recognised the potential role of receptionists in identifying and prioritising such cases, but this was difficult to implement given the competing principle that receptionists should respect patient confidentiality, as emphasised by several GPs.

Patients reported a tortuous journey to specialist assessment if they first made contact with out-of-hours services, walk-in centres, optometrists and, more surprisingly, ED. Although not captured in this qualitative study, our quantitative findings showed that only 55% of people who called an ambulance were transported to ED and 20% sought further advice from a GP. We also found the longest delays were experienced by people who first consulted an optometrist.^21^

Difficulty diagnosing TIA is a well-recognised problem in primary and secondary care.^22^ Although some misdiagnosis is bound to occur following initial presentation, a dilemma raised by GPs was the need to refer all ‘true’ cases without overburdening the TIA clinic with TIA mimics.^23^ Some GPs seemed to be using the ABCD^2^ score as an aid to diagnosis, and so, to decide whether to refer or not. The score was designed to predict risk of stroke following TIA, and although it has some discrimination in diagnosis,^24^ its main use is to prioritise allocation of appointments and to facilitate communication between primary and secondary care.^25^

### Strengths and limitations

The main strength of the study is that it builds on our quantitative findings to provide a deeper understanding of the reasons behind delay from patient and GP perspectives. Previous qualitative work seeking to identify causes of delay has focussed on patients’ accounts. This study provides an ‘insider’ perspective which identifies how the systemic complexity of service provision contributes to delay. Limitations are that it was conducted in a single centre, and at a particular point in time, and that patients without capacity to consent were excluded. Additionally, the only service providers we interviewed were GPs; it would have been helpful to gain insights from others, including GP receptionists and staff in ED and walk-in centres. Inevitably, the study also excluded patients with TIA who either did not seek professional help, and those who were not referred to secondary care.

### Implications for practice and research

The priorities in managing TIA and minor stroke are to admit to hospital if symptoms are still present, and to refer to a TIA clinic using the ABCD^2^ score if symptoms have resolved. This should be achievable whether patients present to ambulance services, general practice, ED or other services, including optometrists.

The focus for ambulance services has been urgent transfer for people with continuing symptoms to maximise chance of thrombolysis,^26^ but it is also important that they are able to arrange direct referrals to a TIA clinic, as advising a GP appointment will introduce unnecessary delay. Since the time of our study, several protocols to enable this have been developed, but there is no national standard. For example in Milton Keynes, the policy is to refer all cases to the TIA clinic,^27^ whereas in East of England, the policy is to refer cases where ABCD^2^ is three or less, and to transport higher risk patients to ED.^28^ Similar direct referral pathways should also be universally available to walk-in, urgent care centres and optometrists. Although direct referral from ED was available at the time of our study, we found it was not always used, emphasising the need for staff training, which may be challenging given rapid turnover.

For general practice, providing a same day appointment and/or identifying patients who need to call an ambulance raises broader issues of receptionist training and/or triages of request for urgent care by a HCP. General practices need to review how they can best handle cases that need an urgent or emergency response, including patients presenting with stroke or TIA. For stroke, modelling has suggested that ensuring all patients who contact their GP are treated as emergencies could increase thrombolysis rates by 16%.^29^ One strategy could be to improve receptionist training,^15^ although this is more likely to be effective for major stroke than TIA. Another strategy could be triaging of all requests for urgent appointments by a nurse or doctor. Although this has been shown not to affect total workload,^30^ its benefits for patient safety have not been examined. Research is also needed to develop tools to help GPs and other front-line professionals to diagnose TIA, using data derived from primary care.^31^
