# Supplementary material for: Service factors causing delay in specialist assessment for TIA and minor stroke: a qualitative study of GP and patient perspectives
Source: BMJ Open. 2016 May 17;6(5):e011654. doi: 10.1136/bmjopen-2016-011654 (PMC4874118; doi:10.1136/bmjopen-2016-011654)
Supplement: Supplementary data [file bmjopen-2016-011654supp.pdf]

Appendix: TOPIC GUIDES FOR QUALITATIVE INTERVIEWS

PATIENTS

Tell me about what happened when you had a TIA / stroke.
What did you notice?
When did you first notice anything? Or if not, when did someone else in the family notice anything? What led up to it?
What was it like?
Who if anyone was around at the time? What was it like for them?
What did you / others *think* had happened? What was it that made you think that?
What did you / others do next?
Was it hard, or easy, to know whether to do anything about it? Can you tell me more about that …..?
So was there a clear decision about needing to get medical help, or were you (others) a bit unsure, or did it just not seem the sort of thing that needed a medic?
What made it clear / made you unsure?
What happened next? (Once a decision was made about getting help, or not).
Probe what led to consulting a particular service – who did they try first (GP, A&E, ambulance etc), what made them choose that avenue, did they think of any others, was it hard or easy to choose who to contact first ….
Did you feel as if anything helped you to get medical attention quickly?
(Probes eg would it have been a different story if: different symptoms, different time of day / week; difference in your own or someone else's medical knowledge, difference in your own or someone elses previous experience of stroke or TIA, different response from services ….)

Did you feel as if anything made a bit of a delay in getting medical attention?
(Probes e.g. would it have been a different story if symptoms had been different, different time of day/week, not worried that family/friends would be burdened by need to give you a lift or other, had different or more information or previous experience, different response from services ….
Explore details of stories of medical encounters (or other relevant events) including referral to stroke specialist, with probing about what felt difficult or complex and what felt straightforward – both decisions and actions .
Looking back is there anything you wish you'd known or done?

Is there anything you are very glad that you (or someone else) did, or knew about?
Any messages you'd like to give to people who find themselves experiencing your symptoms, about what they should do?
Any messages you'd like to give to doctors, nurses or other professionals, about what they should do?

GPs
First, at what stage did you find out about (patient)'s TIA or stroke? (follow up, probe as appropriate – presumable some GPs will not have known until after diagnosis, if patient went straight to hospital in ambulance)
Tell me about what happened when (patient) suffered symptoms of stroke or TIA, so far as you know.
What symptoms did they experience first of all?
Did these symptoms change at all?
Who if anyone was around at the time or don't you know?
What did they do about the symptoms (or what did someone else do) – or don't you know?
To what extent did you suspect a stroke or TIA when they consulted (if they did) – were there factors that made it difficult to decide what the diagnosis might be?
What made it clear / made you unsure?
What happened next? (Once a decision was made about getting help, or not).
Did you feel as if anything helped this patient to get a *quick diagnosis* (if applicable)?
(Probes eg would it have been a different story if: different symptoms, different time of day / week; if they had consulted someone else first, if they had described less clearly (a) particular key symptom(s) ….

Did you feel as if anything made a bit of a delay in *getting diagnosed*?
(Probes e.g. would it have been a different story if symptoms had been different, different time of day/week, etc
Did you feel as if anything helped this patient get *treatment* quickly?
Did you feel as if anything contributed to any *delay* in getting treatment? (remembering this is confidential and we shall not identify individuals in reports)
Explore details of stories of medical encounters (or other relevant events) including referral to stroke specialist, with probing about what was difficult or complex and what was straightforward – both decisions and actions .
Looking back is there anything you wish someone had known or done (eg the patient, a carer, a medical professional?)

Is there anything you are very glad that you (or someone else) did?
Any messages you'd like to give to people who find themselves experiencing those symptoms, about what they should do?
Any messages you'd like to give to other professionals, or the NHS in general, about how to avoid delay in diagnosing or treating TIA and stroke?
